# ‘IT RESTORED A BIT OF THEIR HUMANITY”: BRIDGING THE GAP THROUGH HANDS-ON INTERGENERATIONAL LEARNING

**DOI:** 10.1093/geroni/igac059.2759

**Published:** 2022-12-20

**Authors:** Katherine Meszaros, Renee Beard

**Affiliations:** College of the Holy Cross, Worcester, Massachusetts, United States; College of the Holy Cross, Worcester, Massachusetts, United States

## Abstract

The Covid-19 pandemic laid bare many of the social inequities and vulnerabilities within the American nursing home industry. Intergenerational learning is thought to be an effective approach to debunking ageism and addressing social isolation, especially in institutional care settings. This study used a mixed method approach to explore the benefits of intergenerational learning, here between college-aged students and nursing home residents. We analyzed qualitative observational data, including 56 hours of participant observation during TimeSlips™ sessions with 32 students and 30 residents at a nursing home in Worcester, Massachusetts. A content analysis of 68 student reflections on the classroom community-based learning (CBL) experience was also performed. This poster reports on the findings from the students who participated in the intergenerational learning project in 2020–2022. A series of common themes emerged. Many students began their experience as skeptics, but retrospectively reported that it was transformative. Personal interactions with elders revealed to students the shared humanity that had with older generations, which then forced them to confront their own ageism and ableism. Intergenerational learning also encouraged students to reflect on their lives, ask what type of person they want to be, what world they would like to live in, and gain new life lessons and perspectives on now to age meaningfully. The value of intergenerational learning far exceeds providing nursing home residents with social stimulation. It can be just as formative to the personal philosophies and outlooks of college-aged students. Intergenerational learning provides tangible and intangible “in-the-moment” benefits to those who participate.

